# Does mothers’ postnatal depression influence the development of imitation?

**DOI:** 10.1111/jcpp.12413

**Published:** 2015-04-10

**Authors:** Oliver Perra, Rebecca Phillips, Rhiannon Fyfield, Cerith Waters, Dale F. Hay

**Affiliations:** ^1^ School of Nursing and MidwiferyQueen's UniversityBelfastNorthern IrelandUK; ^2^ Department of PsychologyUniversity of ExeterExeterUK; ^3^ School of PsychologyCardiff UniversityWalesUK

**Keywords:** Learning, infancy, postnatal, maternal depression

## Abstract

**Background:**

Links between mothers’ postnatal depression (PND) and children's cognition have been identified in several samples, but the evidence is inconsistent. We hypothesized that PND may specifically interfere with infants’ imitation, an early learning ability that features in early mother–infant interaction and is linked to memory, causal understanding and joint attention.

**Methods:**

A randomly controlled experiment on imitation was embedded into a longitudinal study of a representative sample of firstborn British infants, whose mothers were assessed for depression using the SCAN interview during pregnancy and at 6 months postpartum. At a mean of 12.8 months, 253 infants were presented with two imitation tasks that varied in difficulty, in counterbalanced order.

**Results:**

The infants of mothers who experienced PND were significantly less likely than other infants in the sample to imitate the modelled actions, showing a 72% reduction in the likelihood of imitation. The association with PND was not explained by sociodemographic adversity, or a history of depression during pregnancy or prior to conception. Mothers’ references to infants’ internal states during mother–infant interaction at 6 months facilitated imitation at 12 months, but did not explain the link with PND.

**Conclusions:**

The findings support the hypothesis that associations between PND and later cognitive outcomes may partly derive from effects of the mother's illness on infants’ early learning abilities. Support for infants’ learning should be considered as an age‐appropriate, child‐focused component of interventions designed to ameliorate the effects of PND.

## Introduction

Links between mothers’ postnatal depression (PND) and children's cognition have been identified in several studies where mothers were diagnosed using clinical interviews and children tested on age‐appropriate tasks (e.g. Azak, 2012; Cogill, Caplan, Alexandra, Robson, & Kumar, 1986; Galler, Harrison, Ramsey, Forde, & Butler, 2000; Hay, Pawlby, Waters, & Sharp, 2008; Hay et al., 2001; Murray, 1992; Murray, Kempton, Woolgar, & Hooper, 1993; Sharp et al., 1995). However, the evidence is inconsistent. The association appears at some ages but not others. For example, in a longitudinal case‐comparison study of families drawn from a population cohort in Cambridgeshire, PND was associated with problems in sensorimotor skills in infancy (Murray, 1992) but not IQ scores in middle childhood (Murray, Hipwell, Hooper, Stein, & Cooper, 1996); however, a sleeper effect emerged, with the adolescent offspring of mothers with PND less likely to do well on national achievement examinations (Murray et al., 2010).

There is relatively consistent evidence for an association between PND and children's sensorimotor skills (Azak, 2012; Galler et al., 2000; Hay et al., 2001; Hay & Kumar, 1995; Koutra et al., 2013; Murray, 1992; Sharp et al., 1995), which may be moderated by the child's sex (Azak, 2012; Sharp et al., 1995) or birthweight (Hay & Kumar, 1995) or SES (Murray, 1992). The cognitive outcomes may be explained by depression in pregnancy (Evans et al., 2012) or the chronic nature of mothers’ illness (Campbell & Cohn, 1997; Cornish et al., 2005) and family stress (Jensen, Dumontheil, & Barker, 2014), but when those factors are controlled, a link between PND and cognitive development is still seen (Hay et al., 2001, 2008).

It is biologically plausible that exposure to the environmental changes linked to PND could have a unique effect on developmental changes in the postnatal brain (Craik & Bialystock, 2006). However, few studies have identified mechanisms whereby mothers’ depressive symptoms following childbirth could exert such effects on cognition, although most have examined features of depressed mothers’ interactions with their infants (e.g. Cohn, Campbell, Matias, & Hopkins, 1990). Mothers’ infant‐focused speech in the months after childbirth is compromised by PND (Murray et al., 1993, 1996). Depressed mothers respond less contingently to infants’ behaviour and their interactions feature more negative affect (e.g. Campbell, Cohn, Flanagan, Popper, & Meyers, 1992; Field, 1992). Hay (1997) proposed that infants’ exposure to less positive affect and lower levels of maternal responsiveness could potentially interfere with the infants’ learning and memory (cf. Dunham, Dunham, Hurshman, & Alexander, 1989; Fagen, Ohr, Fleckenstein, & Ribner, 1985).

In this study, we focus on imitation, a learning ability that develops in infancy (Carpenter, Nagell, Tomasello, Butterworth, & Moore, 1998; Jones, 2009) and is associated with other cognitive skills such as memory (Hayne, Boniface, & Barr, 2000; Rose, Feldman, Jankowski, & van Rossem, 2011), causal understanding (Kiraly, Csibra, & Gergely, 2013) and joint attention (Carpenter, Nagell et al., 1998). Mutual imitation is a characteristic feature of mother–infant interaction (Masur, 1998; Pawlby, 1977). In the presence of PND, the infant's ability to imitate might be reduced. We tested this hypothesis in a controlled experiment embedded within a longitudinal study of a nationally representative sample of firstborn British infants.

Developmental theorists have distinguished *mimicry*, copying the model's action with no regard for its goals, and *emulation*, copying the results of the model's actions with little sensitivity to the model as a social being (Want & Harris, 2002), from true imitation, which entails infants’ sensitivity to the model's intentions (e.g. Carpenter, Akhtar, & Tomasello, 1998); according to the proponents of this ‘social‐cognitive paradigm’, true imitation entails understanding the association between other people's goals and means as well as infants’ ability to reproduce the models’ actions when they pursue the same goal. To examine whether infants could copy the model's actions and not just explore objects the model had used, Carpenter and colleagues compared an *instrumental action* task in which the experimenter manipulated a prominent object, for example a lever, with an *arbitrary action* task in which the experimenter gestured in a noninstrumental way (Carpenter, Nagell et al., 1998). Twenty‐four infants were observed monthly between 9 and 15 months of age. The ability to imitate arbitrary actions emerged slightly later than instrumental imitation, but most infants eventually passed both tasks.

We adapted Carpenter's tasks for use in this study and tested whether infants of mothers with PND might show less imitative learning, especially on the more difficult arbitrary action task. Because of prior evidence that mothers with PND show less positive affect and infant‐focused behaviour (e.g. Murray et al., 1993), we measured mothers’ expression of positive affect and their references to infants’ thoughts and feelings, as possible mediators of any link between PND and infants’ imitative learning.

In testing for effects of PND, we controlled for the mothers’ prior lifetime history of depression. Furthermore, in view of the recently reported associations between mother's depression during pregnancy and children's cognitive outcomes (Evans et al., 2012; Koutra et al., 2013), and the evidence that prenatal stress influences the HPA axis, which in turn affects hippocampal function (Weinstock, 2008), we repeated the analysis controlling for prenatal depression in particular.

## Methods

### Participants

The present experiment was embedded within the Cardiff Child Development Study (CCDS), a prospective longitudinal study of firstborn infants. The CCDS was approved by the UK National Health Service (NHS) Multi‐Centre Research Ethics Committee; 332 first‐time mothers were recruited from antenatal clinics in two NHS Healthcare Trusts in Wales and through a specialized midwifery team designed to support pregnant women at high social risk. Potential participants provided their addresses, including postal codes. Both those who chose to participate and those who chose not to participate in the study represented the entire range of socioeconomic categories associated with UK postal codes. The final sample was nationally representative, not differing significantly from first‐time parents in the most recent UK national cohort study (K. Kiernan, personal communication, April 2009).

During a home visit at a mean of 6.6 months (*SD* 0.9) postpartum, 301 parents were given a semistructured clinical interview and observed with their infants. One parent was interviewed by telephone, eight provided questionnaires, six families had withdrawn from the study, four failed to keep appointments, eight could not be traced within the time window, and four could not be assessed due to ill health or adverse family circumstances.

At the laboratory visit at a mean of 12.8 months (*SD* 1.2), 275 families attended the laboratory session and further 17 provided questionnaire data only. Six further families had withdrawn from the study; 12 cancelled appointments and could not be rescheduled within the timeframe; nine were not traced; and seven could not be assessed at this wave, due to work commitments, poor health or adverse family circumstances.

Of the 275 infants who visited the laboratory, 253 (92%) completed the imitation experiment successfully. Reasons for missing data included failure to complete all tasks (2%); equipment problems (3%); and scoring problems due to camera angles or positioning of the box (3%). Demographic characteristics of the full sample and this subsample are presented in Table 1. The families of infants in the imitative learning experiment did not differ significantly from the full sample on the demographic variables.

**Table 1:** Demographic characteristics of the CCDS sample

Variable	Total sample (*N* = 332)	Imitation task (*N* = 253)
Mother's age at birth (Mean)	28.2	28.7
Stable partnerships	90.4%	90.5%
Marital status (% married)	50.3%	55.3%
Ethnicity (% British or Irish)	92.7%	92.6%
Social class (% middle class)	50.9%	55.3%
Mother's education (% ≥ basic age 16 qualifications)	78.3%	81.0%
Child's sex (% female)	43.3%	45.8%

### Procedure

#### Home visit during pregnancy

Mothers were interviewed during the third trimester of pregnancy, using the Schedules for Clinical Assessment in Neuropsychiatry (SCAN: Wing et al., 1990). Mothers also reported on their educational history, occupation and personal circumstances.

#### Early infancy home visit

One month before the infant entered the age window (range, 5–8 months), a researcher booked the appointment. The visit comprised a second administration of the SCAN and a 30‐min observation of the infant with the primary daytime caregivers (88% mothers).

Three parent–infant interaction tasks were used: free play in which parents were encouraged to play a game they might ordinarily play with their infants; a topic‐sharing task using a commercial activity board; and a feeding task in which the parent fed the infant solid food or, if still exclusively breastfeeding, gave the infant some water on a spoon. Parents’ and infants’ smiling and laughing was recorded across all three tasks for a total of 9 min of observation.

Parental speech to the infant was measured during the topic‐sharing task, a paradigm commonly used in research to elicit conversation between parents and young children (LaBounty, Wellman, Olson, Lagattutta, & Liu, 2008). The activity board depicted cartoon animals, on different flaps like pages in a book. The parent–infant pair sat in a comfortable position on a sofa or on the floor. The parent was told: ‘Show (infant's name) this toy. Take him/her through the pictures’.

#### Late infancy laboratory visit

Infants were assessed individually, in the presence of their caregivers (90% mothers), for 25 min. Because testing was undertaken prior to a simulated birthday party designed to assess peer interaction, three families were tested in separate rooms. If one family was very late and the first infants to arrive showed signs of fatigue, the individual testing was shortened for the infant who arrived late.

The order of the tasks within the battery was randomized. Infants sat on the caregiver's lap in front of a small table in a quiet room decorated with brightly coloured posters. A video camera was placed in one corner of the room approximately 1–1.5 m from the infant.

**Figure 1:**
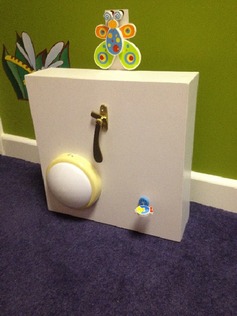
The apparatus used for the imitative learning experiment

Two imitation tasks (adapted from Carpenter, Nagell et al., 1998) were administered in counterbalanced order. A specially designed box was embedded with three objects: a door knocker, a panel light and a spring (Figure 1). In the *instrumental action* task, the model manipulated a structural feature of the box, for example a lever, leading to a contingent outcome. In the *arbitrary action* task, the experimenter made an arbitrary hand movement (e.g. tapping on the surface of the box), which led to a different contingent outcome.

Parents (90% mothers) were given the following instructions: ‘Now we're going to see if babies this age can work out how things work by learning from what another person is doing. This is not a test, so please don't try to help your baby do it. We need to know the age at which babies can learn things in this way, without any help from their parents’.

During the tasks, the experimenter sat beside the parent and infant; the infant remained on the parent's lap. The experimenter explained: ‘I am going to show (infant's name) a box to play with; then I am going to show him/her some ways to play with it. Can I please ask you to let (infant's name) play on his/her own and not to encourage him/her to do anything else than what he/she does on his/her own’.

During a 1‐min baseline period, the infant was given access to the box to play freely. If the infant did not seem interested in the box, the experimenter invited him/her to ‘play with it’. The experimenters recorded the actions and objects used during the baseline. For the instrumental task, the experimenter then modelled an action using an object that had not been manipulated by the infant during baseline (lifting the handle of the door knocker, pressing on the light panel or flicking the spring). This procedure ensured that the infant's imitation represented new learning, not response facilitation of past actions.

Each action was repeated three times, with each demonstration followed by the activation of the puppet. The experimenter showed excitement at the puppet's appearance. After three demonstrations, the experimenter asked the child, ‘Can you do the same?’ The words ‘copy’ or ‘imitate’ were not used. The infant was allowed to play with the box for the 1‐min test period.

For the arbitrary action task (which either occurred immediately after baseline or after the instrumental imitation task, depending on counterbalancing), the experimenter took the box and said to the infant, ‘I am going to show you something new’. The experimenter tapped his or her thumb on a prespecified corner of the box and immediately shook a rattle with the other hand hidden behind the box. This action was repeated three times, with the experimenter showing excitement and asking ‘Did you hear that?’ The box was given back to the infant who was asked to ‘do the same’. The infant was left to play with the box for 1 min. If the child performed any action that involved touching the same location on the box, the experimenter shook the rattle behind the box. If the child performed an action that had been demonstrated during the instrumental task, the experimenter said, ‘Oh, that is gone now’. If the child did not play with the box spontaneously, the experimenter asked the child again to ‘do the same’ but refrained from demonstrating the target action again.

### Measures

#### Mothers’ psychopathology before and after childbirth

The SCAN interviews given in pregnancy and in the early infancy home visit were coded according to DSM‐IV diagnostic criteria. Final decisions about clinical diagnoses were made in case conferences with two adult psychiatrists. In pregnancy, a subset of 33 cases was randomly selected for a reliability sample, revealing significant agreement between the two psychiatrists’ diagnoses of antenatal disorder, *κ* = .79, *p *< .001, and past disorder, *κ* = .81, *p *< .001. At 6 months, mothers were again interviewed using the SCAN, with all possible cases reviewed in case conferences with an adult psychiatrist. Diagnoses of PND were made with good agreement, *κ* = .80, *p *< .001.

Dichotomous variables measured the presence of a DSM‐IV depressive disorder prior to conception, during the pregnancy or in the first 6 months postpartum. The first two variables were combined as a measure of maternal depression prior to childbirth. Three women who met criteria for bipolar disorder were categorized as depressed because the primary feature of their illness was recurrent episodes and symptoms of depression.

#### Sociodemographic adversity

A general index of child's exposure to maternal factors known to be associated with risk for social adversity was created using principal component analysis (PCA). The maternal experiences that contributed to this index were as follows: (a) the mother not having achieved basic educational attainments (i.e. the mother having no qualifications or fewer than five GCSEs or equivalent attainments); (b) the mother being 19 years of age or under at the time of child's birth; (c) the mother not being legally married during the pregnancy; (d) the mother not being in a stable couple relationship during the pregnancy; and (e) the mother's occupation being classified as working class according to the Standard Occupational Classification 2000 (SOC2000; Elias, McKnight, & Kinshott, 1999). All these items were categorical; therefore, the PCA was based on the polychoric correlation matrix. The PCA confirmed that all these items contributed to a single component (eigenvalues 3.84 and 0.68 for the first and second component extracted, respectively); this component explained approximately 77% of the shared variance in these risk indicators. We used summary scores derived from this PCA as a proxy for the family's exposure to socioeconomic adversity.

#### Parent–child interaction

Parents’ and infants’ positive affect (smiling and laughing) during the home visit was coded using 10‐s interval time sampling across the three parent–infant interaction tasks. Independent observers recorded 34% of the videos with good agreement (median *ICC *= .96). A measurement model using maximum likelihood for missing values was used to generate a single factor score. A single factor accounted for approximately 39% of the variance in mothers’ expression of positive emotion (smiling and laughing) across the three different interactive contexts.

The parent's speech during the activity board task was transcribed and separated into temporal units of 5 s (24 units per participant). The proportion of intervals in which parental speech occurred was tabulated. A general measure of parents’ talkativeness was computed by dividing the number of 5‐s time units including speech by the total number of time units. Analysis of a subsample of cases (32%) revealed that coders’ records of parental talkativeness were validated by objective measurement of the mean length of parental utterances recorded by Audacity software, *r* (88) = .72, *p* < .001. Each 5‐s interval in which parents spoke was subsequently coded in terms of references to the infants’ thoughts and feelings (Table 2). Two independent raters coded 10% of the transcripts (*N* = 28) with median agreement *κ* = .90 across categories.

**Table 2:** Coding system for mothers’ references to infants’ mental states

Category	Examples	Code	Cohen's K
*Basic Emotions/Physiology* Love, like, enjoy, hate, dislike, fear, hunger, thirst, pain, arousal	Are you hungry? You like the pretty butterfly. Are you not enjoying this game? Don't worry	E	0.90
*Perception* See, hear, taste, smell, feel	Can you see the cow? Can you feel the fluffy lamb?	P	0.88
*Intentional Agency* Attempt, try, acting to achieve a goal, acting with intent, purposeful acting on an object	What are you after? Are you trying to grab them? Can you open this one next? Were you hitting it?	I	0.77
*Desire* Want, desire, wish, hope, ought, should	Do you want to have a go? Are you hoping it's something to eat?	D	0.92
*Belief* Believe, know, suppose, expect, doubt, suspect	Do you think they're slugs? Do you know what that is?	B	1.00

#### Imitation scoring

The imitation tasks were scored for any imitation across the two tasks; any imitation of the instrumental action; and any imitation of the arbitrary action. Agreement between independent observers as assessed on 25% of cases where the imitation task had been administered was κ = .83, *p *< .001.

## Results

### Incidence of postnatal depression

Of 306 women who were assessed with the SCAN interview, 34 (11.1%) met DSM‐IV criteria for depressive illness in the first 6 months postpartum, which is in line with estimated prevalence rates of 10–13% (e.g. O'Hara & Swain, 1996). Of the 34 women diagnosed with PND, 20 (59%) had experienced at least one prior episode; 17 of those (50% of women with PND) had been depressed in pregnancy. Women with PND had experienced a significantly higher level of socioeconomic adversity, as indicated by their adversity component scores, *M* = .60, compared to nondepressed women, *M* = −.14, *t* (304) = 4.28, *p *< .001.

### Infants’ ability to imitate

Of the 253 infants who completed the task, 174 (68.8%) imitated a modelled action at least once. As expected, the instrumental action task proved easier for the infants, with 157 infants (63.3%) copying at least one of the modelled actions (manipulating the door knocker, light panel or spring). Only 55 infants (22.1%) imitated the experimenter's arbitrary gesture (tapping on the wall of the box). Thirty‐eight (25%) of the infants who imitated during the instrumental task also imitated during the arbitrary task, *κ* = .05.

### Postnatal depression and infants’ success on the imitation tasks

The bivariate correlations of all variables used in these analyses are presented in Table 3. The infants of women who had experienced PND were significantly less likely than other infants to imitate the modelled actions. Only 10 (48%) of the infants whose mothers had been depressed postpartum imitated the model at least once, whereas 160 (70%) of other infants imitated at least one modelled action, *χ* ^*2*^ (1) = 4.52, *p *= .03.

**Table 3:** Univariate associations between maternal variables and infants’ imitation

	Postpartum depression	Prior depression	Socioeconomic adversity	Maternal positivity	Mental state talk	Infant age
Prior depression	.34^a^ ^,^ **					
Adversity	.24***	.24***				
Maternal positivity	−.01	.12+	−.10+			
Mental state talk	−.08	−.09	−.20***	.01		
Infant age	.08	−.04	.14*	−.08	−.04	
Any imitation	−.29^a^ ^,^ *	−.07^a^	.13*	−.05	.15*	.03

^+^ *p *< .10; *p < .05; **p < .01; ***p < .001.

a Tetrachoric correlation.

A logistic regression analysis, conducted after satisfactory checks of assumptions (specification errors, multicollinearity, influential outliers), revealed that the association with PND remained significant after controlling for the infant's gender, age in months, exposure to sociodemographic adversity, and the mother's past history of depressive illness, Wald statistic = 6.25, *p = *.01 (Table 4). PND was associated with a significant 72% reduction in the odds of the occurrence of any imitative act by the infant (*OR* = 0.28; 95% *CI* 0.10–0.76). An association was also found between exposure to socioeconomic adversity and imitation. Compared to children exposed to lower levels of adversity, children exposed to higher levels of adversity were significantly more likely to imitate.

**Table 4:** Prediction of infants’ likelihood of showing any imitation of modelled actions (*N *= 249)

Predictor	Value	B (*SE*)	OR	95%	CI
Gender	Female	Reference	Reference		
	Male	−.25 (.29)	.78	.45	to 1.37
Infant's Age	(Centred at mean)	.02 (.13)	1.02	.79	to 1.31
Socioeconomic adversity	(Standardized)	.45 (.17)	1.57**	1.12	to 2.21
Mother's past	No	Reference	Reference		
Depression	Yes	−.26 (.30)	.77	.43	to 1.40
Mother's postpartum	No	Reference	Reference		
Depression	Yes	−1.27 (.51)	.28*	.10	to .76
Constant		1.19 (.26)	3.30***	1.98	to 5.47

*p < .05; **p < .01; ***p < .001.

*LR χ* ^*2*^ (5) = 12.24, *p* = .03; pseudo‐*R* ^*2*^ = .04.

In view of some prior evidence for significant interactions between the child's gender and the impact of PND (e.g. Hay et al., 2001, 2008), we constructed an interaction term for the PND x gender interaction and tested its association with the binary measure of any imitation across the two tasks. The interaction was not significant and its inclusion did not impact significantly on the model fit.

### PND, infant‐focused speech and imitation ability

The final set of analyses focused on 264 infants who were observed with their mothers during the interaction tasks at the early infancy home visit (the other infants having been observed with primary caregiver fathers). Mothers’ expression of positive affect across the three interaction tasks was not associated with imitation. However, infants who imitated had been significantly more likely to hear mothers’ speech about the infants’ thoughts and feelings 6 months earlier, *t* (217) = −2.29, *p* = .02.

We next tested whether mothers’ tendencies to talk about infants’ thoughts and feelings might explain the association between PND and imitation. In a final logistic regression (Table 5), the control variables of socioeconomic adversity, the mother's past history of depression and the infant's age in months were entered at the first step. The mother's diagnosis was entered at the second step, with the mother's references to the infant's thoughts and feelings entered at the third step. Preliminary checks indicated satisfactory meeting of logistic regression assumptions. Mothers’ infant‐focused speech significantly predicted infants’ imitation but did not explain the fact that infants of depressed women were less likely to imitate. Rather the analysis revealed the independent effects of PND and mothers’ speech about infants’ thoughts and feelings on imitation. PND was associated with a 72% reduction in the odds of the infant imitating (*OR* = 0.28, *95% CI* 0.09–0.84) when controlling for other factors.

**Table 5:** Prediction of infants’ likelihood of showing any imitation of modelled actions. Logistic regression analysis (*N *= 219)

Predictor	Value	B (*SE*)	OR	95%	CI	Wald *χ* ^2^ (df)	Pseudo‐*R* ^2^	LR *χ* ^2^ (df)
Step 1						5.43 (3)	0.02	5.92 (3)
Infant's age	(Centred at mean)	.10 (.14)	1.11	.84	to 1.46			
Socioeconomic adversity	(Standardized)	.58 (.20)	1.79*	1.21	to 2.66			
Mother's past	No	Reference	Reference					
Depression	Yes	−.30 (.33)	.74	.39	to 1.42			
Step 2						5.23* (1)	0.04	11.17* (4)
Mother's past	No	Reference	Reference					
Depression	Yes	−1.28 (.56)	.28*	.09	to .84			
Step 3						7.14** (1)	0.07	19.17** (5)
References to mental states	Total	.16 (.06)	1.17**	1.04	to 1.32			
Constant		.61 (.30)	1.84*	1.03	to 3.30			

*p < .05; **p < .01.

Coefficients presented are those obtained at the final step of the model.

In view of recently reported links between mother's depression during pregnancy and children's cognitive outcomes (Evans et al., 2012; Koutra et al., 2013), we repeated the logistic regression analysis in Table 5 using depression in pregnancy rather than all prior depression as a control variable. There was no significant association between depression in pregnancy and the infants’ overall likelihood of imitation. The inclusion of depression in pregnancy in the model did not remove the effect of PND on infants’ imitation.

## Discussion

The present study adds support to earlier findings of a link between PND and cognitive outcomes (e.g. Cogill et al., 1986; Galler et al., 2000; Hay et al., 2001, 2008; Koutra et al., 2013; Murray, 1992) and supports Hay's (1997) claim that PND might exert specific effects on learning abilities. It was noteworthy that the infants of depressed mothers had particular difficulty with the arbitrary action task, which was designed by Carpenter, Nagell et al. (1998) to measure true imitation of a modelled action, not just emulation of the experimenter's exploration of a physical object (Want & Harris, 2002).

The significant association between PND and imitation was not explained by mothers’ past history of depressive illness or their experience of socioeconomic adversity. The unexpected finding that infants who had experienced adversity were more likely to imitate deserves future replication and investigation on its own right. Many studies of infant imitation in the first year of life have tested relatively small samples of infants from homogeneous backgrounds and thus had limited possibilities of detecting imitation differences across socioeconomic strata.

Successful imitation was not associated with mothers’ expression of positive affect across three mother–infant interaction tasks. However, mothers’ speech about infants’ thoughts and feelings did foster later imitation. This finding is in line with a demonstrated link between mothers’ infant‐focused speech (coded with different operational definitions) and another attainment in early cognitive development, infants’ understanding of the object concept (Murray et al., 1993). In that sample, as in the present study, mothers’ expression of affect did not significantly influence infant cognition. These parallel findings across samples and measures suggest that it is important to move beyond comparisons of depressed and nondepressed mothers and explore the particular dimensions of mother–infant interaction that foster early cognitive abilities.

The study has limitations. Effect sizes are not large. Only a brief assessment of imitation was undertaken. Experimental studies of imitation in smaller samples would typically administer a greater number of trials. There was no attempt to oversample cases at risk for PND and so the cell size for the depressed group was relatively small. Further exploration of associations between PND and early learning would profit from the use of a high risk or clinical sample.

Most importantly, the design of our study, with a home visit at 6 months and a laboratory visit at 12 months, did not allow for the observation of the gradual emergence of mutual imitation in mother–infant interaction that increases in the period between 6 and 12 months of age (Masur, 1998; Pawlby, 1977). Therefore, we could not directly test the possibility that mothers’ imitation of infants teaches infants how to imitate (Jones, 2009). Nevertheless, despite these limitations, our study shows that a counterbalanced experiment on infants’ learning abilities could be successfully embedded in a nationally representative community sample in which clinical diagnoses of PND were made.

The link between PND and infants’ imitation was not explained by mothers’ prior history of depression (including depression in pregnancy). This is not to deny the importance of exposure to mothers’ depression at other times. Antenatal experiences do affect brain development and continued exposure to maternal depression does convey added risk. However, depression after childbirth is a marker for a number of changes in family life which place strain on infants’ early learning environments.

It is quite possible that no single biological or social mechanism explains the consistent evidence for links between postnatal depression and infants’ cognitive attainments identified in this and earlier studies (Galler et al., 2000; Murray, 1992; Murray et al., 1993). Different risk and protective factors may influence different aspects of cognitive development, although it is noteworthy that infant‐focused speech fosters infants’ understanding of the permanence of objects (Murray et al., 1993) and their joint attention skills (Roberts et al., 2013) as well as imitation. In future, it would be helpful to conduct theoretically guided research on early cognitive development in relation to infants’ prenatal and postnatal exposure to maternal depression, testing specific hypotheses about effects on cognitive development, rather than using less helpful, all‐purpose developmental assessments that would not pin down key deficits. More precise understanding about ways in which infants’ learning abilities are compromised by the various environmental correlates of postnatal depression is likely to lead to more infant‐focused intervention strategies.

**Key points**

- Postnatal depression (PND) is sometimes associated with poorer cognitive outcomes for children, but the evidence is mixed.
- Imitation is a common feature of mother–infant interaction that might be disrupted by PND; it correlates with other dimensions of early cognitive development.
- In a nationally representative sample of British first‐time mothers and their infants, infants whose mothers were diagnosed with PND were significantly less likely to succeed on imitation tasks.
- The association between PND and infants’ poorer imitation was not explained by sociodemographic adversity or by the mother's depression during pregnancy or prior to conception.
